# Whole-exome sequencing to identify the cause of congenital sensorineural hearing loss in carriers of a heterozygous *GJB2* mutation

**DOI:** 10.1007/s00405-017-4699-0

**Published:** 2017-08-18

**Authors:** Thomas Parzefall, Alexandra Frohne, Martin Koenighofer, Andreas Kirchnawy, Berthold Streubel, Christian Schoefer, Klemens Frei, Trevor Lucas

**Affiliations:** 1 Department of Otorhinolaryngology, Head and Neck Surgery, Medical University of Vienna, Vienna, Austria; 2 Department for Cell and Developmental Biology, Center for Anatomy and Cell Biology, Medical University of Vienna, Vienna, Austria; 3 Clinical Institute of Pathology, Medical University of Vienna, Vienna, Austria; 4 Department of Otorhinolaryngology, Head and Neck Surgery, Vienna General Hospital (AKH), Waehringer Guertel 18-20, 1090 Vienna, Austria

**Keywords:** Hereditary hearing loss, Non-syndromic hearing loss, Heterozygous *GJB2*, Whole-exome sequencing

## Abstract

Bi-allelic variations in the gap junction protein beta-2 (*GJB2*) gene cause up to 50% of cases of newborn hearing loss. Heterozygous pathogenic *GJB2* variations are also fivefold overrepresented in idiopathic patient groups compared to the normal-hearing population. Whether hearing loss in this group is due to unidentified additional variations within *GJB2* or variations in other deafness genes is unknown in most cases. Whole-exome sequencing offers an effective approach in the search for causative variations in patients with Mendelian diseases. In this prospective genetic cohort study, we initially investigated a family of Turkish origin suffering from congenital autosomal recessive hearing loss. An index patient and his normal-hearing father, both bearing a single heterozygous pathogenic c.262G>T (p.Ala88Ser) *GJB2* transversion as well as the normal-hearing mother were investigated by means of whole-exome sequencing. Subsequently the genetic screening was extended to a hearing-impaired cohort of 24 families of Turkish origin. A homozygous missense c.5492G>T transversion (p.Gly1831Val) in the Myosin 15a gene, previously linked to deafness, was identified as causative in the index family. This very rare variant is not listed in any population in the Genome Aggregation Database. Subsequent screening of index patients from additional families of Turkish origin with recessive hearing loss identified the c.5492G>T variation in an additional family. Whole-exome sequencing may effectively identify the causes of idiopathic hearing loss in patients bearing heterozygous *GJB2* variations.

## Introduction

Hearing loss (HL) is a frequent sensory disorder in humans with an onset at different stages of life. HL occurs in approximately 1:1000 newborns [1] and the number rises to 3.5:1000 in adolescence [2]. A genetic mechanism is causative for the disorder in approximately half of children affected by prelingual HL. In the majority of these cases (70%), HL is not accompanied by further symptoms and is, therefore, termed as non-syndromic (NSHL) [3].

The genetic diagnosis of NSHL remains a challenge in many patients due to the high number of genes involved in the disease. The most common causative gene is *GJB2* in the DFNB1 locus that encodes Connexin 26 (gap junction protein, beta-2), a gap junction protein expressed within the stria vascularis, which regulates cochlear development and is essential for maintenance of active cochlear amplification [4]. Variations in *GJB2* account for up to 50% of all genetically caused NSHL cases in Caucasians [5].

Although variations in *GJB2* almost exclusively cause prelingual autosomal recessive HL, the frequency of heterozygous c.35delG in the newborn hearing-impaired population is over 11% compared to a carrier frequency of below 2% in the normal-hearing Austrian population [6]. Whereas screening within *GJB2* heterozygous patients can also lead to the identification of recessive pathogenic variations in additional HL genes [7], we originally hypothesized here that novel alterations affecting the cochlear gap junction network within DFNB1 may be the cause for this discrepancy. Thus, the index patient examined in this study, carrying the heterozygous c.262G>T (p.Ala88Ser) recessive *GJB2* transversion [8], has been prescreened for changes in upstream regulatory sequences [9], the basal promoter [10] and alternative transcriptional start sites [11] and deletions in* GJB6* [12].

In patients where pathogenic changes in DFNB1 are not found, monosymptomatic HL can be due to genetic defects in any of 90 genes identified to date or alterations in unidentified causative genes [13]. Whereas variations in most non-DFNB1 genes cause hereditary NSHL only in isolated families, some variations have been shown to have a high prevalence in certain populations. For example, pathogenic variations in the *TMC1* gene (encoding the transmembrane channel-like protein 1) are found in more than a third of hearing-impaired Jewish patients of Moroccan ancestry [14]. With the exception of variations in *GJB2*, no such common genetic variants have been identified to date in the Austrian population. Due to practical and financial considerations, routine genetic HL screening in Austria has therefore been confined to screening the coding sequence of *GJB2,* thereby excluding almost 50% of affected individuals from genetic diagnosis and counseling.

Massively parallel DNA sequencing (MPS) allows the comprehensive variation screening of all known deafness genes simultaneously. This high-throughput DNA sequencing method has proven to produce technically reliable results in a time- and cost-efficient manner and is gradually being integrated into the routine genetic diagnostics of Mendelian diseases, including hereditary HL [14–16].

In this study, we applied whole-exome sequencing (WES) on DNA samples from an Austrian family of Turkish origin with monosymptomatic genetic HL containing an index patient bearing a heterozygous c.262G>T pathogenic (p.Ala88Ser) transversion. Extended screening of a hearing-impaired cohort consisting of index patients from 24 families of Turkish origin was subsequently performed to estimate the frequency of the disease-causing variants detected by WES in the Austrian-Turkish HL population.

## Patients and methods

### Subjects

Study patients were recruited at the Department of Otorhinolaryngology, Head and Neck Surgery at the Medical University of Vienna, Austria, as part of an ongoing screening program for hereditary HL. Informed consent was obtained from all patients and the parents of minors and the study protocol was approved by the Ethics Committee of the Medical University of Vienna (approval number: ECS 198/2004, with annual extensions to date). Full medical and family histories were obtained including the age of onset, exposure to noise or ototoxic medications and presence of additional ear-related complaints such as tinnitus or vertigo. Additionally, all participants underwent a clinical ear inspection and pure tone audiometry to determine the type and degree of HL. The degree of HL was defined as mild (20–40), moderate (41–70), severe (71–95) or profound (>95) in dB [17].

### DNA sequencing

Genomic DNA was isolated from frozen peripheral venous blood with a commercial DNA extraction kit (Invisorb blood universal kit 1000, STRATEC Molecular, Berlin, Germany). After screening for *GJB2* variations by traditional Sanger sequencing as previously described [8], a deaf index patient with a heterozygous pathogenic *GJB2* variant and both unaffected parents were selected for trio analysis by WES. Capture libraries were prepared using the Illumina Nextera^®^ Exome Capture Kit (Illumina Biotechnology, San Diego, CA, USA) according to the manufacturer’s instructions. The enriched samples were then sequenced on a HiSeq 2000 (Illumina) device at the core facility of the Medical University of Vienna. The identified target variants were then validated and tested for co-segregation with the disease in the remaining family members and a patient cohort with Sanger-based sequencing.

### Bioinformatics

The resulting reads were mapped to the human reference genome version hg19 using the Burrows–Wheeler read aligner [18]. For variant calling, we applied the Genome Analysis Tool Kit [19]. The variant call format files containing the final single nucleotide, insertion, and deletion variants were further analyzed with the Genomatix GeneGrid interface (Genomatix GmbH, Munich, Germany). Initially, the index patient sample was tested for known deafness-causing variations in all coding sequences and splice sites along with 5 bp of the flanking intronic sequences. Additionally, only variants matching the recessive inheritance pattern of the family were included and variants with an allele frequency in the gnomAD database (http://gnomad.broadinstitute.org) [20] greater than 0.05 were excluded.

*Myo15a* sequence comparisons were made with *Homo sapiens* (NP_057323.3), *Pan troglodytes* (XP_016787798), *Mus musculus* (NP_034992.1), *Rattus norvegicus* (XP_008766109), *Canis lupus familiaris* (XP_536660), *Gallus gallus* (XP_414818), *Xenopus tropicalis* (XP_017952739) *and Danio rerio* (XP_001919593) peptides. Protein sequences were aligned with the constraint-based multiple alignment tool (http://ncbi.nlm.nih.gov/tools/cobalt/cobalt.cgi).

## Results

### Clinical presentation of the index family TAR1

**Fig. 1:**
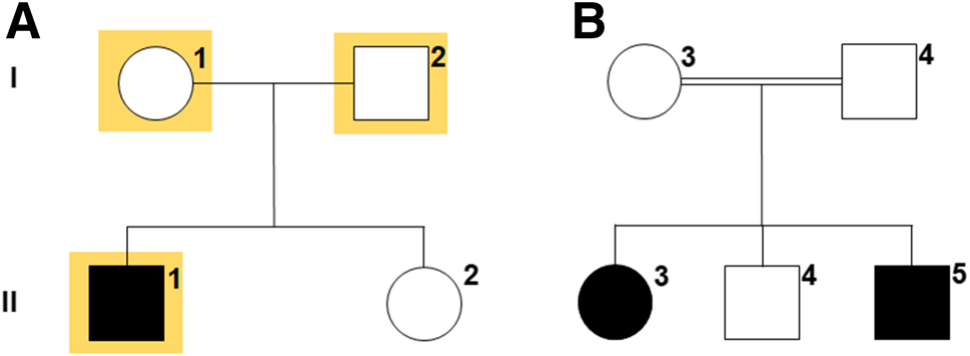
Inheritance in Turkish autosomal recessive families (TAR) suffering from congenital hearing impairment. The index family TAR1 (**a**) comprised a single affected individual (II/1) with a normal-hearing sister (II/2) and parents (I/1 and I/2). Individuals highlighted in *yellow* were selected for whole-exome sequencing. A second family (TAR6) contained two hearing-impaired individuals (II/3 and II/5) with a normal-hearing brother (II/4) and parents (I/3 and I/4). The parents were first cousins (**b**)

The index Turkish autosomal recessive family (TAR1) examined in this study (Fig. 1a) comprised a 7-year-old patient suffering from congenital profound HL (II/1), normal-hearing parents (I/1, I/2) and a normal-hearing sister (II/2).

**Fig. 2:**
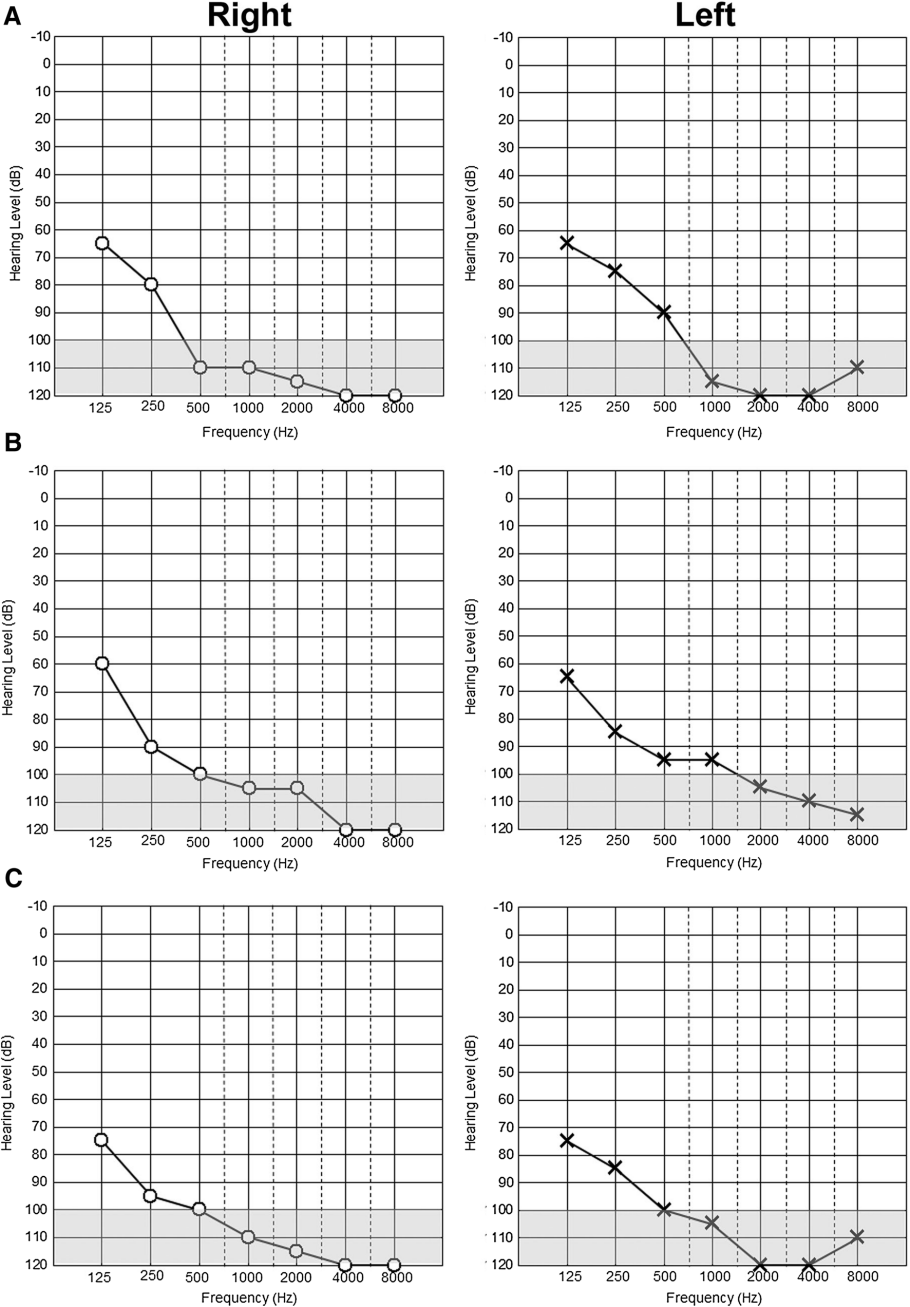
Hearing loss in Turkish autosomal recessive family (TAR) members. Unaided, masked pure tone audiograms in dB hearing loss showing affected TAR1 II/1 (**a**) and TAR6 family members II/3 (**b**) and II/5 (**c**). HL in all cases was severe at frequencies below 500 Hz and mostly profound above 500 Hz

Both the patient and the normal-hearing father were carriers of the heterozygous c.262G>T (p.Ala88Ser) recessive *GJB2* transversion. No additional pathogenic allele within *GJB2* was found in these family members. HL was severe at frequencies below 500 Hz and profound above 500 Hz (Fig. 2a). The patient (II/1) communicates with a combination of severely dyslalic speech, sign language and lip reading. Hearing aids had been supplied since early infancy in Turkey but were insufficient to allow proper language acquisition. Hearing rehabilitation by means of cochlear implantation was not possible due to insufficient auditory pathway plasticity at the age of first presentation at our department. No other symptoms besides HL were observed in the patient.

### Whole-exome sequencing and mapping results

WES was carried out on DNA samples of a deaf index patient (II/1) and both unaffected parents (I/1 and I/2). Sequencing of the capture library of the probands resulted in an average total read number of 6.7 Mbp with a median base coverage of 42×, with 91 and 68% of targeted bases covered by more than 10 or 30 reads, respectively.

### Identification of a homozygous missense variation in Myo15a in family TAR1

**Fig. 3:**
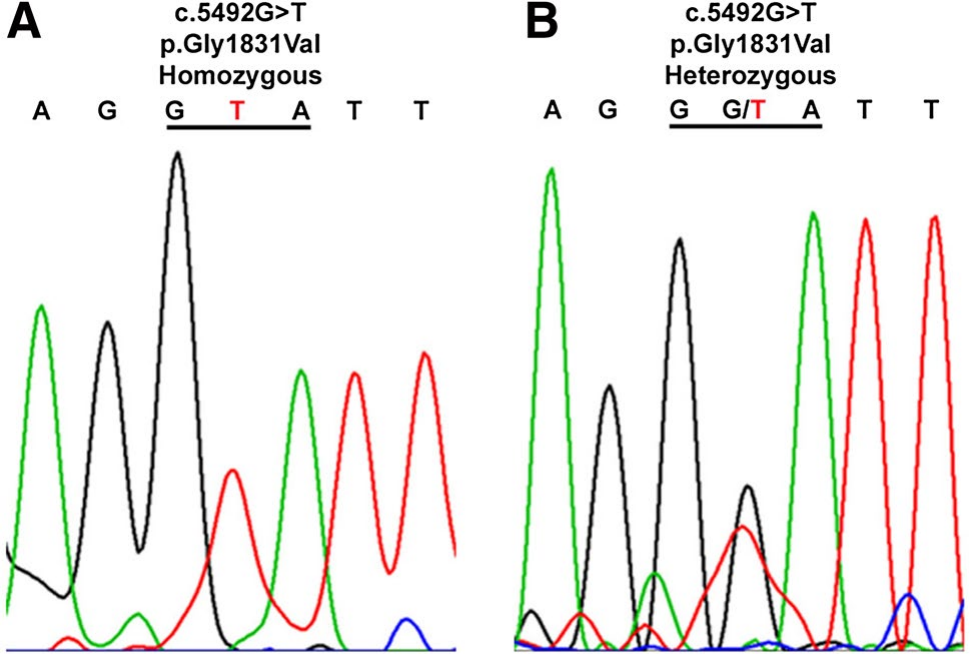
Representative chromatograms showing segregation of the missense c.5492G>T *Myo15a* transversion. In family TAR1, c.5492G>T (p.Gly1831Val) is found homozygous in the affected son II/1 (**a**) and heterozygous in the unaffected mother I/1 (**b**). Codon 1831 is underlined

**Fig. 4:**
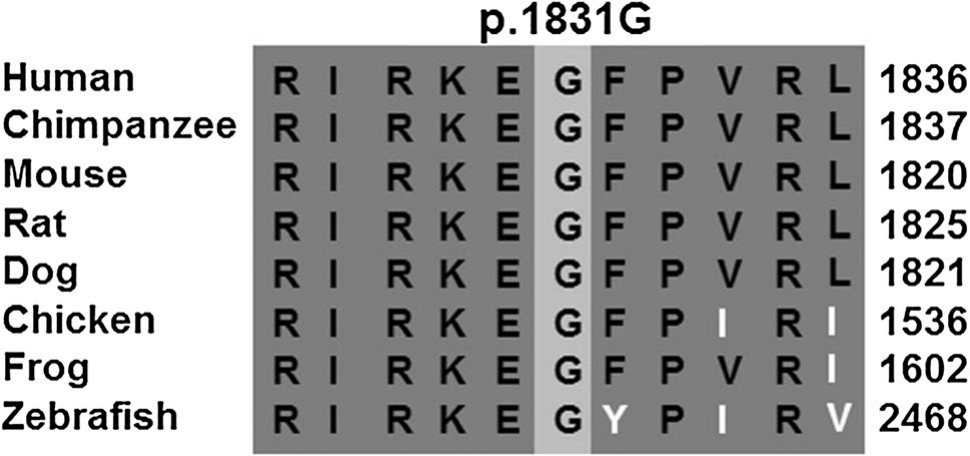
Cross-species multiple alignments of the Myosin 15a peptide. The glycine residue at position p.1831 is highly conserved from *Homo sapiens* to *Danio rerio* sequences

WES of the index family member II/1 identified a homozygous missense G>T transversion in the Myosin 15a gene (*Myo15a*) at position c.5492 (NM_016239) that is predicted to lead to a transposition of glycine to valine at position 1831 of the mature Myosin 15a peptide (p.Gly1831Val). This variant was then validated by Sanger-based sequencing and was found in the affected family member (I/1) in a homozygous state (Fig. 3a). Both parents (I/1 and I/2) and the non-affected sister (II/2) were found to be heterozygous carriers of the c.5492G>T variation (Fig. 3b). This very rare variation, which has previously only been described in a single consanguineous family of Turkish origin, is located at an amino acid position highly conserved throughout evolution from mammals to zebra fish (Fig. 4) and is not listed in the gnomAD database [20, 21].

### Screening for variations in* GJB2* and the c.5492G>T* Myo15a* variation in a cohort of hearing-impaired patients of Turkish origin

Following identification of the c.5492G>T variation in family TAR1, we screened a collective of patients of Turkish origin with recessive inheritance or sporadic incidence of bilateral congenital HL for variations in the *GJB2* gene and for the *Myo15a* c.5492G>T allele to estimate the frequency of this variant in a HL cohort with a Turkish ancestral background (Table 1). In total, 11 familial and 13 sporadic cases with bilateral congenital HL from 24 families were tested. In one additional family (TAR6), 2 further patients with congenital profound deafness were found to be homozygous for c.5492G>T (Fig. 1b: II/3, II/5) whereas both parents (I/3, I/4) and the non-affected sibling (II/4) were heterozygous carriers of the c.5492G>T transversion. The parents were first cousins and the affected siblings II/3 and II/5, aged 19 and 13 years, respectively, suffered from congenital sensorineural HL that was severe at frequencies below 500 Hz and mostly profound above 500 Hz (Fig. 2b, c).

**Table 1:** Overview of the phenotypes and genotypes of representative index patients from each family of the hearing-impaired cohort screened

Index patient	Age	Sex	Phenotype			Genotype	
			Onset	Degree	Progress	*GJB2*	*Myo15a* c.5492G>T
TAR1^a^	7 a	m	con	pro	stable	het c.262G>T	hom
TAR2	8 m	m	con	pro	stable	wt	wt
TAR3	2 a	f	con	pro	stable	wt	wt
TAR4	7 a	f	con	mi-mo	stable	wt	wt
TAR5	8 a	m	con	sev	stable	wt	wt
TAR6	19 a	m	con	pro	stable	wt	hom
TAR7	2 a	m	con	pro	stable	wt	wt
TAR8	3 a	m	con	pro	stable	wt	wt
TAR9	9 a	m	con	sev	stable	wt	wt
TAR10	1 a	f	con	mo	stable	wt	wt
TAR11	1 a	m	con	pro	stable	wt	wt
TAR12	3 a	m	con	mo	stable	wt	wt
TS1	15 a	m	con	mo-sev	stable	wt	wt
TS2	12 a	m	con	sev-pro	progressive	wt	wt
TS3	14 a	m	con	pro	stable	wt	wt
TS4	3 a	f	con	pro	stable	wt	wt
TS5	8 a	f	Post-lingual	mo	progressive	wt	wt
TS6	6 m	m	con	mo	stable	wt	wt
TS7	1 a	m	con	pro	stable	wt	wt
TS8	1 a	m	con	pro	stable	wt	wt
TS9	7 a	f	con	sev	stable	wt	wt
TS10	6 a	f	con	mo	stable	wt	wt
TS11	1 a	f	con	pro	stable	het c.457G>A (benign)	wt
TS12	3 a	m	con	pro	stable	wt	wt
TS13	1 a	f	con	pro	stable	wt	wt

*TAR* Turkish autosomal recessive, *TS* Turkish sporadic, *wt* wild type, *het* heterozygous, *hom* homozygous, *m* months, *a* years, *con* congenital, *mi* mild, *mo* moderate, *sev* severe, *pro* profound

^a^ Index family selected for whole-exome sequencing

## Discussion

In the index family under study, the normal-hearing father and the affected son, suffering from non-syndromic autosomal recessive HL, were carriers of a known c.262G>T pathogenic variant in the *GJB2* gene (Table 1). No second variation within the *GJB2* gene had been identified in previous genetic screening of the patient. Another possible molecular cause for hearing loss in the index patient could have been a digenic inheritance pattern associated with a second variation in the *GJB6* gene that have been described previously [14, 22]. However, digenic *GJB2/GJB6* hereditary hearing loss had been excluded in the patient as described previously [12].

Identification of the homozygous known pathogenic transversion c.5492G>T in *Myo15a* as the underlying causative variation highlights the importance of involving deaf individuals with heterozygous *GJB2* variations in screening for other causative genes. Extending screening for c.5492G>T to a cohort of index patients from 24 families of Turkish descent with congenital HL resulted in a genetic diagnosis in an additional family.

Myosins form a large family of ATP-dependent motor proteins that interact with actin and are essential for a plethora of cellular motility functions. Myosin 15a is a large protein of 3530 amino acids encoded by 66 exons and containing a 1233-residue proline-rich N-terminal domain, an ATPase motor domain, a neck domain with two IQ motifs and a long (1587 amino acids) tail with two myosin tail homology 4 (MyTH4) domains, two band 4.1/ezrin/radixin/moesin (FERM) domains, an Src-homology-3 (SH3) domain and a C-terminal PDZ class I ligand [23, 24].

Myosin 15a is expressed in cochlear outer and inner hair cells and accumulates in the tips of stereocilia. It has been shown to be essential for delivering compounds to the tips that are required for stereocilia elongation during development and for stereocilia maintenance [24–26]. In mice, a variation in the motor domain of Myosin 15a causes the deaf-circling shaker 2 (sh2) phenotype [27] and in humans, variations in *Myo15a* are the cause for DFNB3 type deafness [28]. All known pathogenic variations in *Myo15a* cause prelingual HL [29].

The missense transversion in exon 22 at c.5492G>T (p.Gly1831Val) replaces a highly conserved glycine residue in the Myosin 15a motor domain with valine. Molecular modeling has previously predicted that p.Gly1831Val may inhibit the ability of Myosin 15a to perform the power stroke, which is essential for myosin movement along actin filaments [21].

The *Myo15a* c.5492G>T variant identified is a very rare missense variation so far only identified in a single family with congenital HL in Turkey [21] and is not listed in over 277,000 alleles in the gnomAD database. Our results confirm the pathogenicity of this variant. All three individuals with the homozygous c.5492G>T variation identified in this study and the family described previously show a similar phenotype with profound congenital HL, indicating that this variation has a high penetrance. Families TAR1 and TAR6 had no obvious blood-relationship and none of the family members recalled any positive family history of ancestors that suffered from HL. The occurrence of a homozygous single nucleotide change in three unrelated families can be explained by traditional consanguineous mating in the present population and is most likely the result of a founder allele from a common ancestor. Since the c.5492G>T variation was not present in any of the other 23 families tested in this study, it is likely to play a minor role in the pathogenesis of HL in the general Turkish population.

WES allowed a rapid and cost-effective diagnosis in family TAR1. Traditional homozygosity mapping and candidate gene sequencing would have been a very laborious and costly approach due to the large size of the *Myo15a* gene encoded by 66 exons and would have been of uncertain success due to the small number of available individuals in the index family. The strength of WES in comprehensive screening of large genes and rapid diagnosis even in small families is, therefore, underlined by our results.

## Conclusion

WES and subsequent screening for a known *Myo15a* c.5492G>T (p.Gly1831Val) missense variant provided a genetic diagnosis in 2 of 25 Turkish families with congenital autosomal recessive NSHL. This is the first study to apply WES to genetic deafness screening in Austria. The strength of WES in diagnosing causative variations even in small families and the feasibility to include WES in routine genetic deafness testing in Austria is illustrated by these results. The importance of including patients with heterozygous pathogenic *GJB2* variations in further genetic testing pipelines is also emphasized by this study.
